# Cerebellar Transcranial Alternating Current Stimulation: Frequency-Specific Modulation of Human Gait

**DOI:** 10.1007/s12311-026-02037-8

**Published:** 2026-07-20

**Authors:** Marc Varel, Michael Doppelmayr, Sergiu Groppa, Traian Popa, Manuel Bange, Adrian Zeitner

**Affiliations:** 1 Department of Sport Psychology, Institute for Sport Science, Johannes Gutenberg-University, Mainz, Germany; 2 Department of Neurology, Saarland University Medical Center, Homburg, Germany; 3 MySpace Lab and NeuroRehab Research Center, Service of University Neurorehabilitation (SUN), Lausanne University Hospital, Institution of Lavigny and University of Lausanne, Lausanne, Switzerland; 4 Institute of Computer Science, Informatics for Medical Technology, University Augsburg, Augsburg, Germany

**Keywords:** Cerebellum, Gait, Neuromodulation, Synchronization

## Abstract

Coordinated locomotion depends on rhythmic neural interactions, with the cerebellum contributing to movement timing and scaling. However, how cerebellar oscillatory dynamics influence human gait remains unclear, despite its relevance for motor learning and rehabilitation. We hypothesized two frequency-dependent effects: (i) gait-matched cerebellar transcranial alternating current stimulation (c-tACS) would produce stronger phase alignment than sham and selectively affect temporal rather than spatial gait parameters; and (ii) higher-frequency stimulation would preferentially modulate spatial gait parameters without consistent phase alignment with the ongoing locomotor rhythm. To test this, fifteen healthy adults received randomized bilateral c-tACS at their individual gait-cycle frequency (iGCF), iGCF ± 10% offsets, individual step frequency (iSF), a harmonic in alpha range (iGCF×10), 50 Hz (gamma), and sham. Head-mounted accelerometry recorded kinematics of continuous walking and a stop-and-go task. Phase synchrony between stimulation and gait periodicity was quantified using the debiased phase-locking value (dPLV). 50 Hz c-tACS increased gait velocity and stride length without altering cadence or stride time, indicating selective modulation of spatial gait parameters. In contrast, gait-matched stimulation (iGCF, iSF) produced strong stimulation-gait phase alignment but did not enhance temporal gait parameters beyond sham, arguing against entrainment-specific behavioral effects. dPLV declined with iGCF ± 10% during continuous walking, whereas a transient increase at iGCF-10% during stop-and-go was not specific to active stimulation. These findings support frequency-dependent effects of c-tACS on locomotion and refine the mechanistic understanding of how rhythmic cerebellar stimulation interacts with gait control, with potential implications for targeted neuromodulation.

**Supplementary Information:**

The online version contains supplementary material available at 10.1007/s12311-026-02037-8.

## Introduction

The ability to walk independently is crucial for physical and psychosocial well-being, maintaining quality of life and personal autonomy [1–3]. Conversely, gait impairments have the potential to result in significant disability, particularly among older adults or individuals afflicted with neurological disorders. Preserving or restoring effective gait is therefore vital to reduce the risk of dependency and improve overall health [1, 3–6].

Human locomotion is generated by a hierarchical control system spanning spinal, brainstem, cerebellar, and cortical circuits. Spinal central pattern generators (CPGs) provide the basic rhythmic drive, while supraspinal locomotor regions in the brainstem initiate, modulate, and suppress locomotion, enabling speed-dependent and context-sensitive adjustments of gait [7–9]. These circuits are therefore essential for rhythm generation and execution.

Cerebellar circuits contribute to the patterned control of locomotion by integrating multisensory feedback with predictive motor signals, refining interlimb coordination, postural stability, and step-to-step consistency [2, 10]. Through its interactions with motor cortical areas and the basal ganglia, the cerebellum shapes spatial and temporal properties of an ongoing rhythm [11]. At a computational level, cerebellar contributions to locomotion are commonly framed in terms of predictive control. Internal models implemented within cerebellar circuits encode the expected timing of afferent and efferent events, enabling phase-precise adjustment of ongoing movement [12, 13]. Temporally structured olivocerebellar signalling refines Purkinje cell output, supporting accurate alignment between predicted and actual movement dynamics [14, 15]. Converging evidence indicates that cerebellar activity tracks motor frequency during cyclic actions such as repetitive finger tapping [16] and predicts temporally structured sensory input [17], supporting its role in temporal prediction during ongoing behavior.

Beyond timing, the cerebellum also modulates the gain of motor output. Consensus accounts emphasize its influence on movement amplitude, variability, and vigor through cerebello-thalamo-cortical interactions [18]. Experimental studies further show that cerebellar activity can regulate sensorimotor gain and cortical excitability, particularly at higher frequencies, thereby shaping the spatial features of movement without necessarily enforcing phase alignment [19, 20]. Together, these findings suggest that cerebellar influences on gait may be expressed either through temporally precise rhythm alignment or through modulation of motor gain and spatial gait features, reflecting partly separable modes of cerebellar network function.

Cerebellar transcranial alternating current stimulation (c-tACS) offers a frequency-specific means of probing these computational modes. Applied rhythmic electric fields can interact with ongoing neural activity in a frequency- and phase-dependent manner [21–23]. Depending on stimulation parameters, network state, and frequency, c-tACS may act through entrainment of ongoing oscillations via periodic modulation of membrane potential and spike timing, or through frequency-dependent shifts in cerebellar excitability that alter network gain without stable phase locking [21, 23, 24]. This distinction is important in the present context, because stimulation frequency may bias whether gait effects reflect phase-aligned coupling, excitability-related modulation, or both, thereby complicating mechanistic interpretation.

Consistent with this view, neurophysiological studies demonstrate frequency-dependent effects of c-tACS on cortical excitability and network connectivity, in line with resonance-like properties of cerebello-thalamo-cortical loops [25–27]. Behavioral studies further suggest that c-tACS can influence posture and gait, although reported effects are heterogeneous. Offline and online stimulation protocols have been associated with changes in gait variability, cadence, and turning performance across healthy individuals and clinical populations, with outcomes depending on frequency, task demands, and disease state [28–31]. However, these studies typically investigate only single stimulation frequencies or broad frequency-band contrasts, limiting mechanistic interpretation.

Notably, Koganemaru et al. (2020) provided initial evidence that gait rhythm aligns with online c-tACS at individual gait-cycle frequency (iGCF) compared to sham and skin stimulation during overground walking [32]. While this study suggested phase alignment between cerebellar stimulation and locomotor dynamics, the effects of systematically varying stimulation frequency, including detuned and harmonic conditions, on kinematic gait characteristics and phase synchrony across different locomotor contexts remain unknown.

To address this gap, the present study examined whether c-tACS differentially modulates phase alignment between stimulation and gait, as well as spatio-temporal gait characteristics, as a function of stimulation frequency. Using sham-controlled protocols during continuous walking and gait transitions, we investigated four conceptual stimulation categories selected on behavioral and physiological grounds: gait-matched, detuned, alpha-range harmonic, and fixed gamma conditions (see Methods). Based on the findings of Koganemaru et al. (2020), and on the cerebellum’s established role in predictive movement timing [32] and precise encoding of rhythmic input [16, 17], we hypothesized that gait-matched c-tACS would produce stronger phase alignment than sham, consistent with entrainment-like coupling, and selectively affect temporal rather than spatial gait parameters. Because gait transitions engage distinct locomotor demands – including anticipatory postural adjustments and re-initiation of rhythmic movement – we further examined whether such effects differed between continuous walking and the stop-and-go task. In contrast, higher-frequency stimulation was expected to preferentially modulate spatial gait parameters without producing consistent phase alignment with the ongoing locomotor rhythm [20].

During continuous walking and a stop-and-go task, head-mounted accelerometry combined with bilateral heel pressure sensors quantified gait kinematics, including left-right gait-event timing. Phase relationships between the stimulation waveform and locomotor rhythm were assessed as a behavioral index of temporal alignment grounded in phase-dependent coordination frameworks [33]. Together, these measures were designed to capture both the temporal and spatial dimensions of stimulation-gait coupling across frequency conditions and locomotor contexts.

Demonstrating that stimulation frequency differentially affects stimulation-gait phase alignment and kinematic gait characteristics would provide causal insight into cerebellar contributions to rhythmic locomotor control and could inform the development of frequency-tailored tACS protocols for gait rehabilitation.

## Methods

### Participants

An a priori power analysis was conducted using G*Power (Heinrich Heine Universität, Düsseldorf, Germany, v.3.1.9.7) and indicated a required sample size of *N* = 7 (*f* = 0.51, *α* = 0.05, 1–*β* = 0.95, seven within-subject conditions, *r* = 0.5). With *N* = 15, the design was sensitive to detect effects of *f* ≥ 0.29 (*ηp*² ≥ 0.08), corresponding to medium within-subject magnitudes (details in supplemental information).

Fifteen healthy adults (6 females; mean age = 25.1 ± 2.3 years) participated. Mean body height was 176.5 ± 8.8 cm, body weight 75.8 ± 11.3 kg, corresponding to a mean BMI of 24.3 ± 2.9 kg/m² and an individual gait-cycle frequency of 0.92 ± 0.05 Hz (mean ± SD; Table 1). 

**Table 1:** Participant demographics and baseline gait frequency

Subject	Sex	Handedness	Age	Body height	Body weight	BMI	
1	male	right	28	194	89	23.65	0.84
2	male	left	23	186	87	25.15	0.88
4	female	right	25	170	80	27.68	0.96
5	female	right	26	174	62	20.48	0.99
6	male	right	23	187	86	24.59	1.00
7	male	right	23	178	73	23.04	0.95
8	male	right	21	178	68	21.46	0.89
9	female	right	27	162	78	29.72	0.93
10	male	right	26	179	72	22.47	0.93
11	male	right	23	172	61	20.62	0.88
12	female	right	28	175	85	27.76	0.85
13	male	right	23	186	93	26.88	0.91
14	male	left	24	176	84	27.12	0.91
15	female	left	28	165	64.7	23.76	0.97
		***M***	25.07	176.47	75.85	24.31	0.92
		***SD***	2.29	8.77	11.32	2.91	0.05

Sex, handedness, age, body height and weight, body mass index (BMI), and individual gait cycle frequency (iGCF) derived from baseline walking. Subject 3 was excluded from mean (M) and standard deviation (SD)

Exclusion criteria held a history of neurological, cardiovascular, or untreated psychiatric disorders. Handedness was assessed using the Edinburgh Handedness Inventory, identifying 12 right-handed and 3 left-handed participants. Hand dominance was not considered critical, given the bilateral stimulation and the largely symmetric cerebello-cortical motor connectivity across hemispheres [34], consistent with prior evidence that gait control is minimally influenced by handedness [35].

Participants reported an average of 7 h 15 min of sleep before testing, with a self-rated sleep quality of 7 on a 9-point Likert scale (1 = very poor, 9 = very good). All participants provided written informed consent prior to participation. The study was conducted in accordance with the Declaration of Helsinki and approved by the ethical review committee of the State Medical Association of Rhineland-Palatinate (Germany).

### Study Design

#### The study employed a randomized, double-blind, sham-controlled design.

A 5-minute baseline walk was recorded prior to stimulation to determine the individual gait-cycle frequency (iGCF), which served as the reference for all subject-specific protocols. Seven stimulation conditions were then completed in randomized order (Table 2), organized into four conceptual groups to systematically test cerebellar responsiveness. 

**Table 2:** Stimulation frequencies across protocols.

tACS protocol		iGCF	+10%	-10%	x10	iSF	50 Hz	SHAM
frequency	*M*	0.92	1.02	0.83	9.26	1.85	50	-*
	*SD*	0.04	0.05	0.04	0.46	0.09	.0	-

Group mean and standard deviation (SD) stimulation frequencies across conditions. Order of conditions was randomized. The 50 Hz protocol was not individualized, thereby zero distribution. *Sham consisted of iGCF stimulation ramped up/ -down for 30 s without sustained current

*Gait-matched* – (1) iGCF and (2) individual step frequency (iSF): These conditions used frequencies directly derived from the participant’s gait and step cycles to probe temporally aligned entrainment, building on the initial evidence of rhythm modulation reported by Koganemaru et al. (2020) [34].

*Detuned* – (3) iGCF+10% and (4) iGCF-10%: These protocols applied modest shifts from the base gait frequency to assess the cerebellum’s sensitivity to small frequency deviations – a critical factor for assessing the robustness of entrainment and its relevance for gait rehabilitation.

*Alpha-range harmonic* – (5) iGCF×10: A frequency corresponding to approximately ten times iGCF was applied to target the alpha range. This harmonic condition was included to test whether frequency-specific effects extend beyond the fundamental locomotor frequency into a cerebellar band implicated in movement timing and coordination [25].

*Fixed gamma* – (6) 50 Hz: Because cerebellar gamma-band activity has been linked to sensorimotor network interactions [20], and 50 Hz cerebellar stimulation has been shown to alter cerebello-motor excitability [19], the fixed gamma condition was included to probe effects on spatial gait parameters. 

This design allows temporally specific phase-alignment effects to be distinguished from higher-frequency modulation of spatial locomotor output, potentially related to gain-like mechanisms. Sham stimulation (7) served as a control. 

**Fig. 1:**
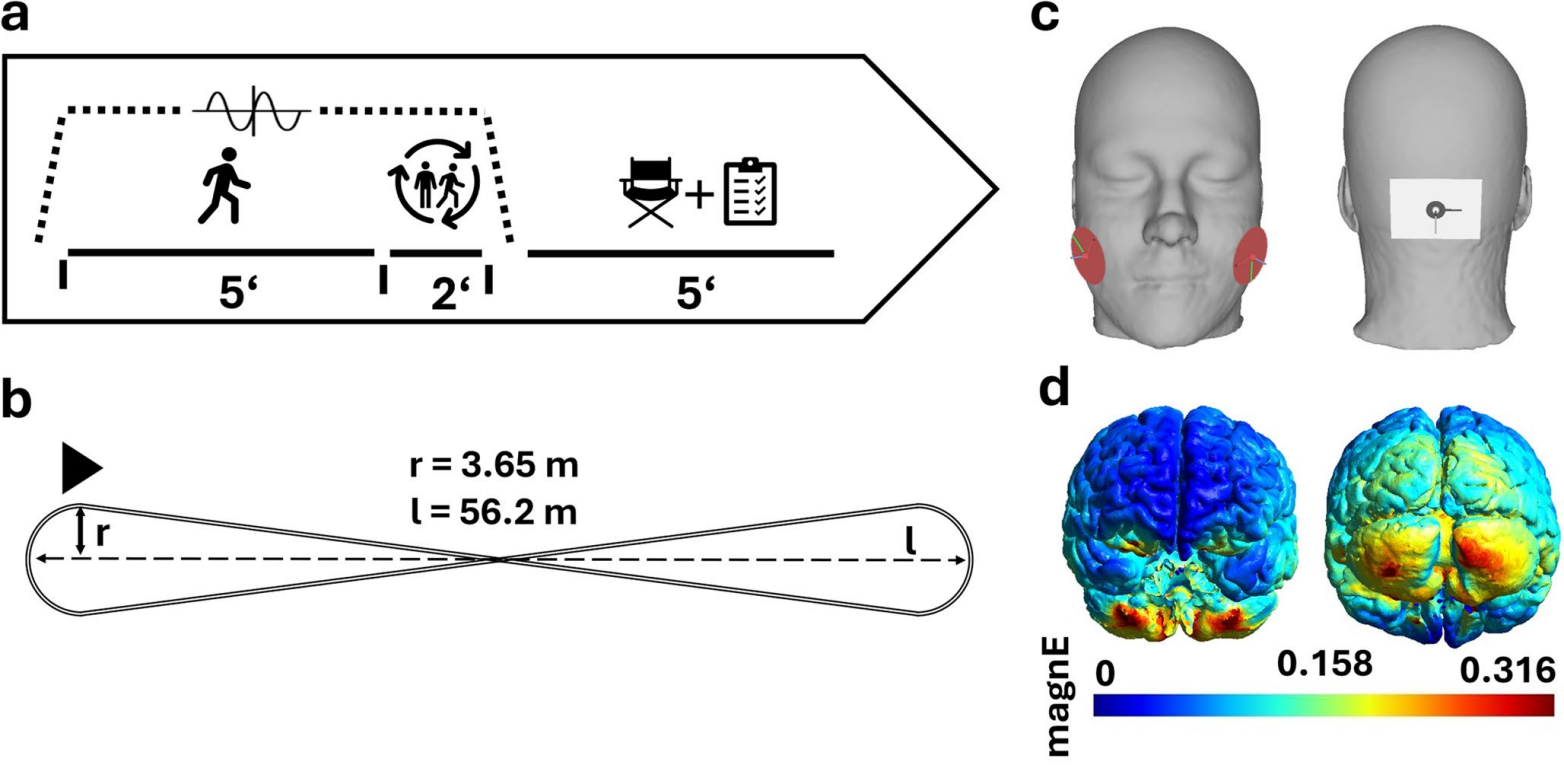
Experimental protocol, walking path, and cerebellar tACS montage with simulated electric field. **a** Task timeline. Following gait initiation, stimulation ramped up over 30 s (dotted line), was held at full intensity during 5 min of continuous walking and a subsequent 2-min stop-and-go task (≥ 10 short sequences), and then ramped down over 30 s. A rest period of ≥ 5 min followed, during which participants completed subjective ratings while seated. Pictograms denote continuous walking, the stop-and-go task, and seated rest with ratings. b Walking path. a figure-eight loop with a turning radius of r = 3.65 m and a distance of l = 56.2 m between the two most distant points of the track, giving a total length of 122.5 m per round. The triangle marks the starting position and walking direction. **c** Stimulation montage. The midpoint of the active electrode (white; 5 × 7 cm) was positioned 2 cm inferior to the inion, targeting the cerebellar midline. Two return electrodes (circular, red; 5.64 cm diameter) were placed bilaterally over the left and right *m. buccinator*. **d** Finite-element model of the resulting electric field magnitude (magnE, in V/m), shown from anterior (left) and posterior (right) perspectives and colour-coded from 0 (blue) to a maximum of 0.316 V/m (red)

Each c-tACS protocol lasted approximately 8 min and comprised a 30 s ramp-up, 5 min of continuous walking, a 2-min stop-and-go block, and a 30 s ramp-down (Fig. 1a, see Montage and stimulation properties). Walking was performed on a figure-eight track (122.5 m per lap; Fig. 1b), minimizing rhythmic distortions associated with sharp turns and preserving natural gait [36]. During the continuous walking block, participants walked at their self-selected comfortable pace, held consistent across conditions; gait parameters were subsequently normalized to each participant’s baseline, controlling for inter-individual differences in preferred pace. 

The subsequent stop-and-go block, initiated by the first auditory stop cue, was included to assess c-tACS effects during gait transitions – specifically distinguishing stimulation effects on locomotor initiation from those observed during steady-state walking. During this block, participants responded to each auditory cue by stopping, briefly standing with their feet together, and resuming walking voluntarily, completing at least 10 stop–restart sequences per condition. Throughout all conditions, participants maintained a steady gaze in the walking direction to ensure consistent visual orientation and minimize head motion artifacts.

Between conditions, participants rested in a seated position for a minimum of 5 min. Resting-state EEG (not analyzed in the present study) and questionnaires assessing subjective perception and tolerability were collected during these intervals. Including preparation, task blocks, and breaks, each session lasted 3–4 h. 

### Transcranial Alternating Current Stimulation

#### Montage and Stimulation Properties

Bilateral c-tACS was delivered through three saline-soaked sponge electrodes (Fig. 1c). The active electrode (5 × 7 cm; 35 cm²; current density: 0.057 mA/cm^2^) was positioned 2 cm below the inion, and currents were returned via two circular electrodes (Ø 5,64 cm; 25 cm²; current density: 0.04 mA/cm^2^) placed over the left and right *m. buccinator* [42]. This montage was selected based on combined computational and experimental work indicating strong cerebellar field engagement with reduced peripheral side effects [43].

Stimulation and signal acquisition were controlled through the Starstim8 device (Neuroelectrics, Barcelona, Spain), ensuring internal synchronization, while recorded signals were transmitted wirelessly via Bluetooth. Stimulation was applied at 2 mA peak-to-peak [41]. Each protocol involved ~ 7 min of stimulation (5 min walking; 2 min stop-and-go). Impedance was monitored and maintained below 10 kΩ; stimulation automatically ceased if values exceeded 20 kΩ. While c-tACS is typically applied for ~ 20 min, we adopted shorter, task-coupled stimulation to maximize mechanistic specificity and feasibility, particularly for future clinical studies in populations with physical limitations. Prior work has shown that c-tACS primarily exerts online effects without robust plastic carryover, supporting this state-dependent approach [25]. Cerebellar stimulation was performed in compliance with current safety recommendations for transcranial electrical stimulation [44].

Individual stimulation frequencies (iGCF, iSF, ± 10% offsets, iGCF×10, 50 Hz) were derived from 5-minute baseline gait recordings, excluding the first minute to avoid habituation-related cadence decline (Table 1). A designated investigator computed stimulation parameters, while data collection was performed by a separate blinded experimenter to ensure double-blind control. Sham stimulation mimicked tACS at iGCF by applying only 30 s ramp-up/-down phases without sustained current delivery [45]. Post-session questionnaires assessed blinding effectiveness.

For phase-alignment analyses, a subject- and condition-specific reference stimulation waveform was reconstructed offline from the programmed stimulation parameters (see Data analysis). 

#### Electrical Field Simulation

The electrode configuration was optimized prior to experimentation using finite element method simulations to estimate the electric field distribution. Simulations were performed with SimNIBS [37, 38] on the standard “ernie.msh” head model, which provides realistic tissue segmentation [39].

Tissue conductivities were assigned according to established values for the direct current range: white matter (0.15 S/m), gray matter (0.4 S/m), cerebrospinal fluid (1.79 S/m), eyeballs and scalp (0.33 S/m), and skull (0.008 S/m) [40]. Electrodes were modelled with a 1 mm conductive layer and 3 mm sponge layer (conductivity = 1 S/m).

Electric fields were computed under the quasistatic approximation [38], simulating the peak excitatory phase of the applied waveform (Fig. 1d). A total of + 2.0 mA was applied to the cerebellar electrode and returned equally (−1.0 mA) through each buccinator electrode, ensuring current balance. The normal component of the electric field within gray matter was extracted from the current density vector, normalized by gray matter conductivity [41].

### Data Acquisition

#### Gait Kinematics

All signals were acquired through the Starstim8 system (Neuroelectrics, Barcelona, Spain) under internally synchronized timing. Gait kinematics were recorded at 100 Hz with a three-axis accelerometer positioned on the EEG cap at the back of the head. Left and right heel strikes were identified using sheet-type pressure sensors attached to the heels and recorded at 500 Hz using NIC2 software (Neuroelectrics, Barcelona, Spain). The combined accelerometer-pressure approach validated previously [46, 47] provides reliable timing of gait events despite the accelerometer’s cranial placement. Prior work demonstrated consistent temporal coupling between peak vertical acceleration and pressure-sensor-defined initial contacts across participants.

EEG was recorded simultaneously through the same system; in the present study, the Oz channel was used only to verify temporal correspondence of the reconstructed stimulation waveform used for phase-alignment analyses.

#### Behavioral Metrics

For each 5-min continuous overground walking sequence, the total distance covered was recorded to characterize spatiotemporal gait performance. Four continuous-walking parameters were derived: gait velocity (m/s), calculated as walking distance divided by walking time; cadence (steps/min), defined as the number of heel strikes per minute; stride length (m), obtained by dividing walking distance by the total number of strides; and stride time (s), computed as total walking time divided by stride count. These measures were extracted exclusively from the continuous walking condition.

During the 2-min *stop-and-go* blocks, three additional behavioral outcomes were analyzed. Self-determined standing time was defined as the interval between the auditory stop cue and gait initiation onset, identified in vertical acceleration via zero-crossing and threshold-based detection. Gait initiation time corresponded to the latency from gait initiation onset to the first acceleration peak indicating heel-strike. Step time was defined as the interval between the first two consecutive heel strikes following gait resumption. All behavioral metrics were derived from synchronized accelerometer and heel-pressure sensor recordings, ensuring precise event identification and cross-validation across modalities.

#### Subjective Perception and Adverse Effects Assessment

After each protocol, participants completed a standardized questionnaire evaluating their subjective experiences. Confidence in being stimulated versus sham was rated on separate 9-point Likert scales (1 = “certainly not/weak,” 9 = “certainly/strong”). Beliefs about whether tACS caused or could cause performance improvements were assessed using the same scale. In this context, performance was intentionally left unspecified to avoid cueing participants toward particular expectations through predefined dimensions such as movement ease, speed, or responsiveness, and instead to capture their overall experience of the stimulation session. Adverse effects, including pain, fatigue, or other physical discomforts, were likewise rated, alongside self-reported concentration during and after stimulation (Fig. 5). Additional side effects could be reported in an open-response field.

### Data Analysis

#### Acceleration Signal Preprocessing and Gait-Event Extraction

Calibration was performed using least-squares offset correction to normalize the mean signal magnitude to gravitational acceleration. Signals were band-pass filtered with a second-order Chebyshev Type II filter (0.25–40 Hz) to preserve gait-relevant frequency content while attenuating out-of-band noise (Fig. S1b). For temporal alignment with heel-strike markers obtained from the foot pressure sensors, the filtered accelerometer signal, originally sampled at 100 Hz, was subsequently resampled to 500 Hz. Downsampling the higher-resolution foot pressure data would have risked losing individual strike events.

The y-axis of the head-mounted accelerometer captured gait-related vertical head acceleration. Gait events were identified by adaptive detection of negative peaks in this signal and validated against heel-pressure markers to assign left and right contacts (Fig. S1a). For phase-synchrony analyses, these events were used to reconstruct a continuous sinusoidal representation of the locomotor cycle (Fig. S1b). Depending on the stimulation condition, phase was defined either by stride (left-to-left heel strikes; iGCF, iGCF ± 10%, iGCF×10, 50 Hz) or by step frequency (left-to-right heel strikes; iSF). For stride-based reconstruction, negative peaks of the reconstructed waveform corresponded to left heel strikes and positive peaks to right heel strikes; for step-based reconstruction, each heel strike was represented by a negative peak, with a positive peak interpolated at the midpoint between consecutive negative peaks.

#### Stimulation-Gait Phase Alignment

The stimulation waveform was not recorded as a separate hardware trace. For each participant and condition, a reference c-tACS waveform was reconstructed in MATLAB (The MathWorks Inc., R2024b, USA) from the stimulation frequency, current amplitude, and timing profile, and used as the phase reference for synchrony analyses. To verify temporal correspondence, the reconstructed waveform was compared with the stimulation artifact in the concurrently recorded occipital EEG-channel, located close to the stimulation site, solely to confirm timing consistency of the phase reference. Following Koganemaru et al. (2020), sham was included in the dPLV analysis using a reconstructed reference waveform at iGCF despite the absence of continuous oscillatory stimulation between ramp phases, to allow comparison of phase alignment estimates between active and sham conditions under an identical analytical framework [32].

Phase synchrony between the c-tACS and the reconstructed sinusoidal gait waveform was quantified using the debiased phase-locking value (dPLV). It measures the consistency of instantaneous phase differences while correcting for sample-size bias. We used the standard bias correction

$$\:dPLV=\frac{N{R}^{2}-1}{N-1},$$

with $\:R=\left|\:\frac{1}{N}\sum\:_{t=1}^{N}{e}^{i\left({\phi\:}_{stim\left(t\right)}-k{\phi\:}_{gait\left(t\right)}\right)}\right|$ the mean resultant length and *\:N* the number of samples per bin. This follows prior discussions of PLV bias [48] and bias-free estimation principles from the pairwise phase-consistency (PPC) framework [49].

Instantaneous phases for the stimulation current $\:{\phi\:}_{\mathrm{s}\mathrm{t}\mathrm{i}\mathrm{m}\left(t\right)}\:$and the reconstructed sinusoidal gait signal $\:{\phi\:}_{\mathrm{g}\mathrm{a}\mathrm{i}\mathrm{t}\left(\mathrm{t}\right)}$ were obtained from the analytic signal of the Hilbert transform applied to the respective filtered time series. Phase differences were adjusted for the stimulation harmonic factor for iGCF×10 and 50 Hz as $\:{\Delta\:}{\phi\:}_{t}={\phi\:}_{stim\left(t\right)}-{k\phi\:}_{gait\left(t\right)}$ (iGCF×10: *\:k=10* for; 50 Hz: $\:k=\:50/\mathrm{i}\mathrm{G}\mathrm{C}\mathrm{F}$).

dPLV was calculated for the steady-state stimulation period, comprising the 5-min continuous-walking segment and the 2-min stop-and-go segment, with the 30 s ramp-up/-down intervals excluded. Gait cycles were detected via zero-crossings of the vertical acceleration and grouped into non-overlapping bins of five gait cycles, ensuring identical temporal resolution between continuous-walking and stop-and-go tasks. This binning procedure preserved local temporal structure within each stimulation block rather than collapsing it into a single estimate. For the stop-and-go task, only locomotor segments were included; standing periods between gait bouts were excluded from phase-synchrony analysis. Descriptive inspection of bin-wise dPLV time courses during continuous walking (Fig. S2) did not reveal a clear systematic within-block trend, supporting the use of condition-level summary estimates. For each bin, dPLV was computed as

$$\:dPLV=\text{}\frac{N\cdot\:{\left|\:\frac{1}{N}\text{}\sum\:_{t=1}^{N}{e}^{i\left({\phi\:}_{stim\left(t\right)}-{k\phi\:}_{gait\left(t\right)}\right)}\right|}^{2}-1}{N-1}\text{}\text{}\text{}$$

Because PLV and its debiased variant are bounded between 0 and 1, and their magnitude depends on the signal-to-noise ratio and the number of analyzed samples, no universal thresholds exist for classifying synchrony as strong or weak. Values were therefore interpreted comparatively across conditions rather than by absolute magnitude, following recommendations for bias-corrected phase coupling metrics [48, 49]. Before averaging, outliers were removed within each subject and condition across all bins using the 1.5 × IQR criterion, with extreme values replaced by *NaN*.

For each subject × condition, the remaining dPLV values were summarized via bootstrap resampling (2000 iterations). To ensure comparable effective sample sizes across conditions, the resample size was defined subject-wise and separately for each task as the minimum number of valid bin-wise observations available across that subject’s conditions after preprocessing and outlier removal. In each iteration, the subject-specific number of valid bins was resampled with replacement from the corresponding subject × condition dataset, and the bootstrap mean was used for inferential statistics.

### Statistical Analysis

One participant was excluded for non-compliance with task instructions (Table 1, subject 3). Outliers were identified parameter-wise using the 1.5 × interquartile range criterion. Final sample sizes are reported in the corresponding figures. 

Spatiotemporal gait metrics (velocity, cadence, stride length) were baseline-corrected (e.g., Δvelocity = protocol - baseline) to account for inter-individual variability. Protocol effects on Δ-values were assessed with repeated-measures ANOVA. When Mauchly’s test indicated sphericity violations, Greenhouse-Geisser corrections were applied, and Tukey-Kramer post hoc tests were used for pairwise contrasts. Stop-and-go metrics (standing time, gait initiation time, step time) were analyzed analogously within the baseline-subtracted ANOVA framework.

For dPLV, statistical analyses were performed on one summary estimate per subject × protocol × task derived as described above. A two-way repeated-measures ANOVA (Condition × Task) was applied with Greenhouse-Geisser correction and Holm-Bonferroni-adjusted post hoc tests. We report partial η² with 95% confidence intervals from the noncentral-F distribution, along with partial ω² as a bias-adjusted effect size. Significance was set at *α* = 0.05 (two-tailed).

Subjective ratings of stimulation perception, performance enhancement, concentration, pain, and fatigue were analyzed using Friedman tests, followed by Dunn-Šidák corrected Wilcoxon signed-rank tests where appropriate. All analyses were conducted in MATLAB (The MathWorks Inc., R2024b, USA).

Full pairwise statistics, confidence intervals, and effect sizes are reported in Supplementary Information.

## Results

### Gait Characteristics

#### Continuous Walking

Across protocols, baseline walking exhibited the lowest average gait velocity (1.38 ± 0.12 m/s), cadence (110.84 ± 5.62 steps/min), and stride length (1.51 ± 0.09 m), except for stride time (1.09 ± 0.06 s). By contrast, 50 Hz stimulation produced the highest mean velocity (1.46 ± 0.12 m/s), stride length (1.55 ± 0.10 m) and cadence (112.56 ± 4.92 steps/min), except for stride time (1.07 ± 0.05 s). The shortest stride length occurred during the iGCF-10% protocol (1.48 ± 0.11 m). Variability represented by the coefficient of variation (CV) was greatest in the SHAM condition across three out of four parameters (velocity CV = 9.75%, cadence CV = 5.56%, stride length CV = 7.79%; Table 3). Stride time yielded the highest CV in iGCF×10 (CV = 5.69%). 

**Table 3:** Descriptive gait parameters across stimulation protocols

		BL	iGCF	+10%	−10%	SHAM	x10	50 Hz	iSF
Gait velocity (m/s)	*M*	1.38	1.40	1.41	1.40	1.42	1.45	1.46	1.44
	*SD*	0.12	0.13	0.13	0.12	0.14	0.12	0.12	0.13
	CV	8.56	9.60	9.41	8.55	9.75	8.45	8.23	8.75
Δ Gait velocity (m/s)	*M*		0.01	0.02	0.02	0.05	0.03	0.07	0.06
	*SD*		0.08	0.08	0.07	0.07	0.08	0.08	0.07
Cadence (steps/min)	*M*	110.84	111.74	111.86	111.94	111.69	112.27	112.56	112.31
	*SD*	5.62	5.63	5.76	5.29	6.21	4.50	4.92	4.78
	CV	5.07	5.03	5.15	4.72	5.56	4.01	4.37	4.26
Δ Cadence (steps/min)	*M*		0.49	0.87	0.92	1.39	0.89	1.76	1.39
	*SD*		2.56	2.45	1.83	2.48	2.34	2.98	2.51
Stride length (m)	*M*	1.51	1.51	1.51	1.48	1.52	1.54	1.55	1.53
	*SD*	0.09	0.11	0.11	0.11	0.12	0.11	0.10	0.11
	CV	5.75	7.15	7.14	7.58	7.79	6.97	6.57	7.29
Δ Stride length (cm)	*M*		0.34	0.87	1.05	2.65	1.02	5.22	4.00
	*SD*		4.19	4.39	5.52	5.37	5.85	5.01	5.11
Stride time (s)	*M*	1.09	1.08	1.08	1.08	1.07	1.08	1.07	1.07
	*SD*	0.06	0.06	0.06	0.05	0.05	0.06	0.05	0.04
	CV	5.37	5.48	5.51	4.98	4.37	5.69	4.45	4.16
Δ Stride time (ms)	*M*		−9.03	−12.00	−13.10	−19.07	−11.82	−21.71	−19.17
	*SD*		20.68	20.23	17.15	24.16	17.59	23.65	22.48

Mean (M), standard deviation (SD), and coefficient of variation (CV) are shown for gait velocity (*n* = 12), cadence (*n* = 14), stride length (*n* = 10), and stride time (*n* = 14) during continuous walking under each stimulation condition. Δ-values represent changes from individual baseline (BL). All values are averaged across participants after outlier removal. Gait velocity and stride length increased most prominently under 50 Hz and iGCF×10 protocols, whereas cadence and stride time remained stable across condition

**Fig. 2:**
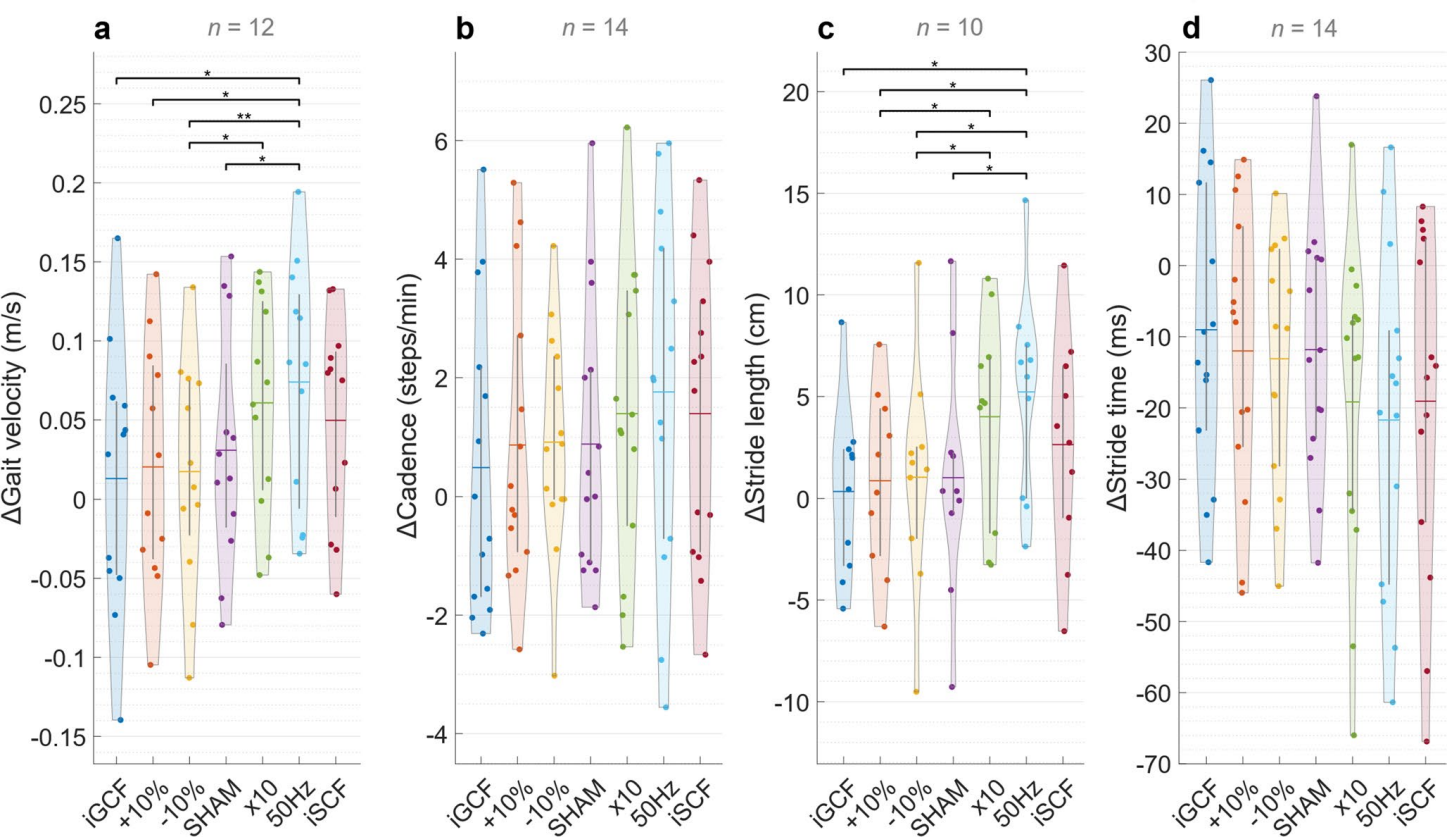
Continuous walking parameters across stimulation protocols. Violin plots show baseline-subtracted (Δ) values for **a** gait velocity (m/s), **b** cadence (steps/min), **c** stride length (cm), and **d** stride time (ms) across all protocols. Sample sizes after outlier removal are indicated above each plot. Each dot represents one participant, and horizontal bars denote group means. The shaded violin curves illustrate the distribution of individual values across participants. * p < 0.05; ** p < 0.01

Analysis of Δ-values relative to baseline revealed a statistically reliable main effect of protocol on gait velocity (F_6, 66_ = 7.80, *p* < 0.001, $\:{\eta\:}_{p}^{2}$ = 0.41 [95% CI 0.20–0.54], $\:{{\upomega\:}}_{p}^{2}$ = 0.36), reflecting a large within-subject effect. Post hoc tests demonstrated that 50 Hz stimulation increased velocity more than iGCF (*p* = 0.007, *d* = 0.76), iGCF+10% (*p* = 0.012, *d* = 0.71), iGCF-10% (*p* < 0.001, *d* = 0.76) and SHAM (*p* = 0.049, *d* = 0.57; Fig. 2). The iGCF×10 protocol also increased velocity compared to iGCF-10% (*p* = 0.017, *d* = 0.62) and showed a statistical trend compared to iGCF+10% (*p* = 0.074, *d* = 0.56). In contrast, gait velocity compared to baseline did not differ significantly among SHAM, iGCF, iSF, iGCF+10%, and iGCF-10%.

Stride length showed a similar pattern (*F*_6, 54_ = 9.05, *p* < 0.001, $\:{\eta\:}_{p}^{2}$ = 0.50 [95% CI 0.27–0.62], $\:{{\upomega\:}}_{p}^{2}$ = 0.44), again representing a large effect. The 50 Hz condition increased stride length significantly more than iGCF (*p* = 0.003, *d* = 1.06), iGCF+10% (*p* = 0.013, *d* = 0.92), iGCF-10% (*p* = 0.005, *d* = 0.79) and SHAM (*p* = 0.024, *d* = 0.77; Fig. 2). The iGCF×10 protocol also lengthened strides relative to iGCF+10% (*p* = 0.010, *d* = 0.66) and iGCF-10% (*p* = 0.035, *d* = 0.56). SHAM, iGCF, iSF, and ± 10% of iGCF produced no significantly different changes. ∆Cadence (F_6, 78_ = 1.02, *p* = 0.395, $\:{\eta\:}_{p}^{2}$ = 0.11 [95% CI 0.02–0.26], $\:{{\upomega\:}}_{p}^{2}$ = 0.04) and ∆stride time (F_6, 78_ = 2.03, *p* = 0.141, $\:{\eta\:}_{p}^{2}$ = 0.13 [95% CI 0.04–0.30], $\:{{\upomega\:}}_{p}^{2}$ = 0.08) were statistically unaffected by stimulation, consistent with a small to moderate effect. Average cadence remained between 111 and 113 steps/min and stride time between 1.07 and 1.09 s across all protocols. 

Gamma-frequency stimulation increased stride length and velocity without affecting cadence and stride time, indicating selective modulation of spatial gait parameters. A smaller enhancement under the iGCF×10 protocol suggests limited facilitation at harmonic alpha frequencies, whereas other conditions produced no significant kinematic effects.

#### Stop-and-Go Task

**Fig. 3:**
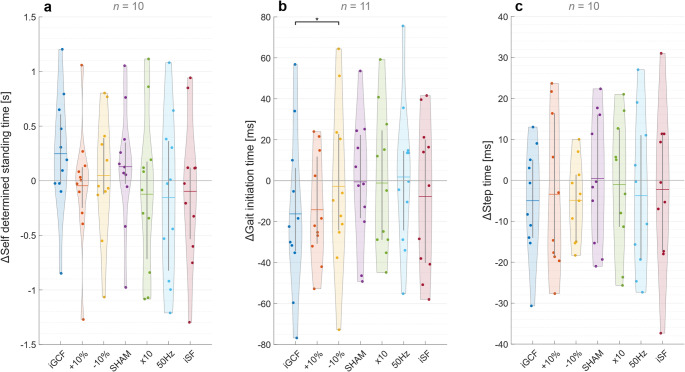
Stop-and-go behavioral metrics across stimulation protocols. Violin plots show baseline-corrected (Δ) values for **a** self-determined standing time (s), **b** gait initiation time (ms), and **c** step time (ms). Sample sizes after outlier removal are indicated above each plot. Each dot represents one participant, and horizontal bars denote group means. An asterisk (*) marks an exploratory post hoc comparison between iGCF and iGCF-10% that did not survive correction for multiple comparisons

Analyses of baseline-corrected behavioural metrics during the 2-minute stop-and-go block revealed no significant main effect of stimulation protocol on Δself-determined standing time (F_6, 60_ = 2.32, *p* = 0.121, $\:{\eta\:}_{p}^{2}$ = 0.19), Δgait-initiation time (F_6, 60_ = 0.88, *p* = 0.470, $\:{\eta\:}_{p}^{2}$ = 0.08), or Δstep time (F_6, 54_ = 0.42, *p* = 0.778, $\:{\eta\:}_{p}^{2}$ = 0.05; Fig. 3). An exploratory post-hoc contrast indicated a decrease in gait-initiation time for iGCF compared to iGCF-10% protocol (*p* = 0.039, *d* = 0.34), but this effect did not survive correction for multiple comparisons and should be interpreted as provisional (Table S3). All other pairwise contrasts were non-significant and effect sizes were small to negligible.

No protocol reliably affected standing time, gait initiation, or step time during the stop-and-go task. The nominal reduction in gait initiation time for iGCF relative to iGCF-10% did not withstand correction, indicating that c-tACS did not produce consistent behavioral changes in gait initiation or termination under the current parameters. 

### Stimulation-Gait Phase Alignment (dPLV)

#### Continuous Walking

Phase-locking between the applied c-tACS waveform and gait exhibited expected frequency-dependent modulation. During continuous walking, iGCF and SHAM produced the highest mean dPLV values with low interindividual variability (iGCF = 0.961 ± 0.048, CV = 4.99%; SHAM = 0.969 ± 0.039, CV = 4.06%), followed by iSF (0.867 ± 0.125, CV = 14.36%). Detuned conditions showed substantially lower dPLV and greater variability (iGCF+10% = 0.547 ± 0.168, CV = 30.68%; iGCF-10% = 0.467 ± 0.094, CV = 20.07%). The iGCF×10 and 50 Hz gamma protocols yielded near-zero dPLV (CV > 100%), indicating an absence of phase synchronization with the locomotor rhythm (Table 4).

**Table 4:** Debiased phase-locking values (dPLV) by protocol and task

	Walking			Stop-and-go task		
	M	SD	CV	M	SD	CV
iGCF	0.961	0.048	4.986	0.933	0.059	6.292
+10%	0.547	0.168	30.681	0.523	0.155	29.649
-10%	0.467	0.094	20.07	0.734	0.106	14.399
SHAM	0.969	0.039	4.058	0.939	0.051	5.426
x10	0.	0.	> 100%	0.	0.	> 100%
50 Hz	0.	0.	> 100%	0.	0.	> 100%
iSF	0.867	0.125	14.362	0.885	0.077	8.643

Arithmetic mean (M), standard deviation (SD), and coefficient of variation (CV) of dPLV during continuous walking and stop-and-go tasks across all stimulation protocols. dPLV was computed from phase differences between accelerometer signals aligned to heel-strike markers and the sinusoidal tACS waveform (see Methods)

**Fig. 4:**
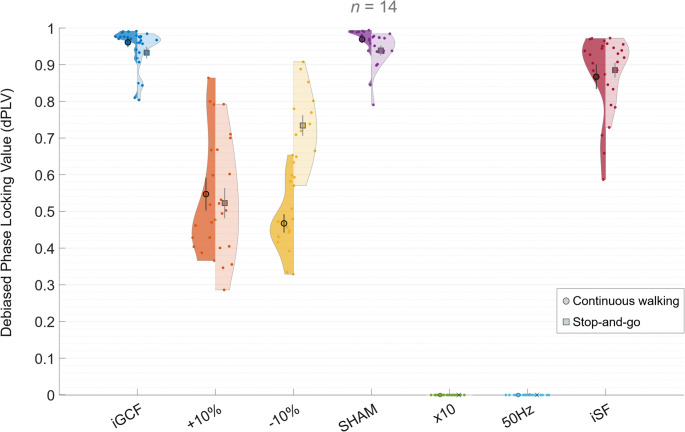
Frequency-specific phase alignment during continuous walking and stop-and-go task. Split-violin plots of bootstrapped dPLV across stimulation conditions during continuous walking and stop-and-go tasks. Each pair of violins shows the distribution of participant means for one stimulation protocol (iGCF, iGCF+10%, iGCF-10%, SHAM, iGCF×10, 50 Hz, iSF). Solid circles denote the group mean ± SEM (vertical bars) for continuous walking, and squares denote the mean ± SEM (vertical bars) for the stop-and-go task. The figure illustrates the frequency dependence of gait-stimulation phase synchrony, with maximal dPLV for gait-matched (iGCF, iSF) and SHAM conditions and near-zero coupling for iGCF×10 and 50 Hz stimulation

A two-way ANOVA revealed a significant main effect of protocol on bootstrapped dPLV values (F₁,₁₃ = 9073.6, *p* < 0.001, $\:{\eta\:}_{p}^{2}$ = 0.999 [95% CI 0.996–0.999], $\:{\omega\:}_{p}^{2}$ = 0.998), confirming that phase synchrony between gait and c-tACS varied substantially across protocols. Holm-Bonferroni-corrected post hoc comparisons revealed three distinct levels of dPLV across stimulation conditions. During continuous walking, iGCF, SHAM, and iSF produced the highest dPLV values and did not differ significantly from one another (*p* > 0.05, Fig. 4). Detuned conditions (iGCF+10%, iGCF-10%) yielded significantly lower dPLV than iGCF, SHAM, and iSF (all adjusted *p* < 0.05) but did not differ between themselves (*p* > 0.05). The iGCF×10 and 50 Hz protocols resulted in near-zero dPLV and were significantly lower than all other conditions, including the detuned protocols (all adjusted *p* < 0.001). Effect sizes for all significant contrasts were very large (*d* > 8), often reaching extreme values (*d* > 30), indicating robust differences within this task. 

#### Stop-and-Go Task

During the stop-and-go task, dPLV again showed a clear frequency-dependent pattern broadly similar to continuous walking (Table 4). The strongest phase-locking was observed in the gait-matched and control conditions, with similarly high values for SHAM (0.939 ± 0.051, CV = 5.43%), iGCF (0.933 ± 0.059, CV = 6.29%), and iSF (0.889 ± 0.077, CV = 8.64%). Detuned conditions showed lower and more variable phase-locking (iGCF+10% = 0.523 ± 0.155, CV = 29.64%; iGCF-10% = 0.734 ± 0.106, CV = 14.40%), whereas the iGCF×10 and 50 Hz protocols produced near-zero dPLV values (CV > 100%), indicating no consistent phase alignment with the locomotor cycle. The similarly high dPLV values in SHAM again suggest that elevated phase-locking at gait-matched frequencies primarily reflects intrinsic gait periodicity rather than stimulation-specific entrainment.

A similar three-level pattern compared to continuous walking emerged from the ANOVA. SHAM, and gait-matched conditions again exhibited the strongest phase-locking, without significant differences among them (*p* > 0.05). Detuned conditions (iGCF+10%, iGCF-10%) produced intermediate dPLV values that were significantly lower than SHAM, iGCF, and iSF (all adjusted *p* < 0.05) but did not differ from each other (*p* > 0.05). As in continuous walking, iGCF×10 and 50 Hz stimulation produced near-zero dPLV and were significantly lower than all other conditions (all adjusted *p* < 0.001). Across both tasks, SHAM remained comparable to gait-matched stimulation, while iSF tended to yield slightly lower dPLV than iGCF and SHAM yet remained significantly higher than detuned and high-frequency protocols. Effect sizes were uniformly very large within both tasks, underscoring the stability of the frequency-dependent hierarchy of phase synchrony across conditions.

#### Continuous Walking vs. Stop-and-Go Task

A significant main effect of task was observed (F₁,₁₃ = 6.60, *p* = 0.023, $\:{\eta\:}_{p}^{2}$ = 0.337 [95% CI 0.002–0.631], $\:{{\upomega\:}}_{p}^{2}$ = 0.272), indicating that walking task (continuous vs. stop-and-go) modulated the strength of phase-locking. Across tasks, post hoc comparisons confirmed that iGCF-10% stimulation and iGCF×10 exhibited significant task effects (both *p* < 0.05). Specifically, the iGCF-10% condition showed higher dPLV during stop-and-go compared with continuous walking, while the iGCF×10 protocol yielded minimal yet statistically detectable task differences. No other protocol demonstrated a significant difference between tasks (all *p* > 0.05).

The interaction between condition and task did not reach significance (F₁,₁₃ = 3.82, *p* = 0.072, $\:{\eta\:}_{p}^{2}$ = 0.227 [95% CI 0.002–0.632], $\:{{\upomega\:}}_{p}^{2}$ = 0.158), though a trend toward task-dependent modulation was evident. 

Phase synchrony between gait and cerebellar stimulation exhibited pronounced frequency dependence and moderate task sensitivity. Strong phase-locking occurred at gait-matched frequencies (iGCF, iSF) and under SHAM, whereas detuned protocols (iGCF+10%, iGCF-10%) significantly reduced synchronization (Fig. 4). iGCF×10 and gamma 50 Hz stimulation produced near-zero dPLV, suggesting that phase alignment is frequency-specific and aligned with the locomotor rhythm rather than imposed by the stimulation. Stop-and-go walking elicited slightly higher coupling in the iGCF-10% condition and minor differences for iGCF×10, while phase-locking patterns were otherwise consistent across tasks. Collectively, these results confirm that gait-matched c-tACS interacts with the intrinsic temporal structure of gait (Table 4).

### Subjective Perception and Adverse Effects Assessment

**Fig. 5:**
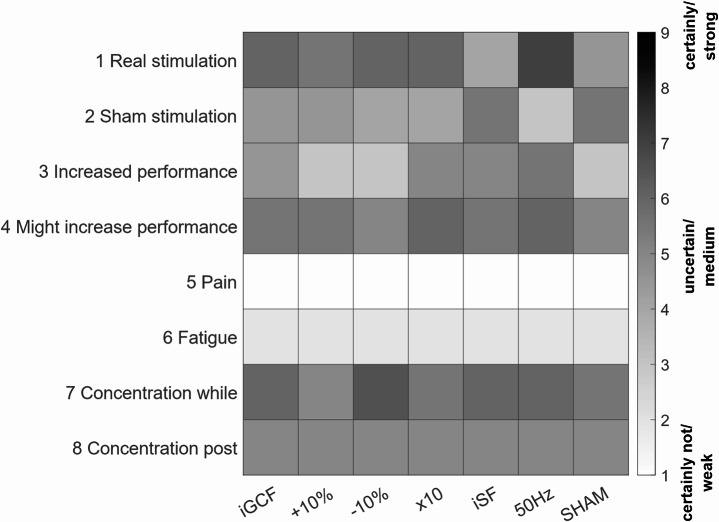
Subjective perception and tolerability of c-tACS. Median ratings are displayed for stimulation perception, sham perception, perceived performance enhancement, concentration, pain, and fatigue. Participants rated each domain on a 9-point Likert scale (1 = certainly not/weak, 9 = certainly/strong)

After each protocol, participants rated perceived stimulation, sham perception, performance enhancement, concentration, pain, and fatigue on a 9-point Likert scale (Fig. 5). A Friedman test revealed significant differences in ratings of *being stimulated* (χ²(6) = 22.977, *p* < 0.0001, *W* = 12.347) and *being sham stimulated* (χ²(6) = 17.117, *p* = 0.009, *W* = 13.132). Post-hoc Wilcoxon signed-rank tests with Dunn-Šidák correction confirmed that only the 50 Hz condition was rated higher than SHAM (*p* < 0.05). Specifically, 50 Hz was perceived as real stimulation (median = 7, IQR = 2) compared to SHAM (median = 3, IQR = 2). Ratings for all other protocols clustered near SHAM, suggesting participants were generally unable to distinguish them.

The perceived performance enhancement varied but without robust post hoc effects. A main effect of condition was observed (χ²(6) = 13.79, *p* = 0.032, W = 13.40), but none of the pairwise comparisons reached significance. Ratings of pain and fatigue remained low and stable across protocols, and concentration levels were reported as moderate to high with only minor variability.

These findings indicate that subjective differentiation between stimulation protocols was limited, with 50 Hz perceived as the most salient condition but not associated with increased adverse effects. Overall, bilateral c-tACS was well tolerated across all conditions.

## Discussion

The present findings provide partial support for frequency-dependent effects of cerebellar stimulation on gait: gamma-range stimulation modulated spatial gait output, whereas gait-matched stimulation primarily reflected phase alignment without evidence for entrainment-specific behavioral facilitation. Specifically, 50 Hz c-tACS increased gait velocity and stride length without affecting cadence or stride time, whereas gait-matched frequencies produced robust stimulation-gait phase coupling but did not yield selective changes in temporal gait characteristics.

Consistent with the prediction for higher-frequency stimulation, 50 Hz c-tACS was associated with increased gait velocity and stride length without corresponding changes in cadence or stride time, suggesting an effect on spatial rather than temporal gait characteristics. In contrast, gait-matched frequencies produced high dPLV without evidence of behaviorally relevant entrainment effects. Because stimulation frequency was derived from each participant’s gait rhythm, elevated dPLV values were expected and indeed observed during both continuous walking and stop-and-go conditions. Importantly, however, dPLV values under sham were comparable to those observed during gait-matched stimulation (Table 4), indicating that the observed phase locking likely reflects the inherent rhythmic regularity of locomotion rather than stimulation-induced synchronization. We therefore interpret these findings as temporal alignment between the stimulation waveform and gait rhythm [50], inferred from behavioral phase metrics [24].

In line with the absence of entrainment effects, gait-matched stimulation did not yield selective behavioral facilitation during continuous walking: iGCF, iSF, and sham elicited similarly modest changes in velocity, cadence, stride length, and stride time relative to baseline (Table 3). Moreover, entrainment would be expected to reduce stride-to-stride variability, which was only descriptively apparent and not statistically supported. These findings also help contextualize prior work. Koganemaru et al. (2020) reported c-tACS-related modulation of gait rhythm, which was interpreted as an entrainment effect. Yet, their paradigm involved repeated turning, which intermittently perturbed steady-state gait rhythm and may have increased sensitivity to external rhythmic input [32]. By contrast, the present overground figure-eight protocol minimized such turn-related perturbations and evaluated continuous locomotion separately from a stop-and-go task to test whether entrainment-like effects differed by locomotor context. However, the present data did not support such effects.

Notably, iSF did not yield stronger phase alignment despite the bihemispheric montage, suggesting that bilateral stimulation alone does not enhance step-level coupling. Although the buccinator-return montage was chosen based on modelling, experimental and tolerability work [43], the electric field is still anatomy-dependent, potentially limiting laterality- and frequency-specific effects. Future gait-matched c-tACS protocols should test more field-model-guided designs.

In contrast to the low-frequency conditions and consistent with our hypothesis, gamma c-tACS was associated with increased gait velocity and stride length without altering cadence or stride time, indicating effects on spatial gait parameters rather than locomotor timing. Although statistically reliable, these effects were small in absolute magnitude in this cohort of healthy young adults, and their functional significance remains uncertain. Mechanistically, however, this pattern is more consistent with modulation of cerebellar output state than with phase-aligned temporal coupling [51]. One possible substrate is altered Purkinje-cell regulation of the deep cerebellar nuclei, which project via cerebello-thalamo-cortical and cerebello-brainstem pathways to influence the amplitude of descending motor commands [10, 18]. This interpretation is supported by evidence linking gamma-range cerebellar activity to sensorimotor network interactions [19], and gamma-frequency stimulation to changes in cerebello-motor physiology, including motor cortical excitability [20, 28].

Phase alignment showed sharp frequency selectivity: although gait-matched conditions exhibited high dPLV, even modest detuning (± 10%) markedly reduced coupling. While progressive cycle-to-cycle phase drift under detuned stimulation inevitably lowers dPLV, genuine entrainment-like adaptation would have counteracted this by shifting walking cadence – or equivalently decreasing stride time – toward the stimulation frequency, thereby sustaining phase coherence across the analysis window. Neither was detected. The dPLV decrease therefore reflects the absence of locomotor frequency entrainment and confirms the physiological relevance of the detuning manipulation rather than a purely mathematical analysis artefact. Watanabe et al. (2026), applying cerebellar tACS at identical detuning levels, similarly reported differential spatiotemporal effects without cadence convergence toward the stimulation frequency [52]. Together, these findings are consistent with the Arnold Tongue framework, in which phase locking of a driven oscillator declines steeply beyond a narrow entrainment bandwidth [27, 53, 54].

Task context had comparatively modest effects: dPLV was slightly higher during stop-and-go than continuous walking, primarily driven by the iGCF-10% condition, indicating that gait initiation may transiently enhance susceptibility to phase alignment. However, this effect likely reflects kinematic transients rather than genuine neural coupling, as gait initiation engages distinct control mechanisms relative to steady-state walking [55, 56] and typically involves transient slowing and longer step times [57, 58]. Such transitions may represent windows of increased sensitivity to rhythmic perturbation, consistent with transition-locked stimulation approaches reported in Parkinson’s disease [59].

The iGCF×10 protocol yielded small but significant increases in stride length and gait velocity relative to the detuned (± 10%) conditions, while no differences were observed relative to sham and phase locking remained negligible. These findings argue against locomotor phase entrainment at this frequency. Instead, stimulation in the alpha range may have weakly biased cerebellar timing-related network dynamics, consistent with evidence linking low-frequency cerebellar activity to temporal prediction and coordination [25, 60–62].

Taken together, the present results refine the interpretation of c-tACS effects during locomotion. 50 Hz stimulation was associated with modest increases in gait velocity and stride length despite negligible stimulation-gait phase coupling, indicating a dissociation between spatial gait effects and phase alignment under the present conditions. Although small in absolute magnitude, these spatial changes may still be mechanistically informative, because they indicate selective sensitivity of stride-related parameters under a frequency-specific stimulation condition despite the relative stability of gait in healthy young adults. In contrast, gait-matched stimulation did not provide evidence for selective modulation of temporal gait properties. Although iGCF and iSF produced high dPLV values, their comparability to sham suggests that they primarily reflected intrinsic gait periodicity rather than stimulation-specific entrainment. Evidence for additional effects of the alpha-range harmonic (iGCF×10) on combined timing-related and spatial gait processes was limited under the present conditions.

Several limitations should be considered. The present results are restricted by the use of healthy young adults and a single-session experimental design. The high temporal stability of gait parameters observed in this cohort (Fig. 2b), together with strong stimulation-gait alignment in the gait-matched conditions, likely reflects an already well-optimized locomotor control system and may have limited the behavioral modulation by c-tACS. Future studies should prioritize populations with less stable or pathological gait, in whom the locomotor system may be more susceptible to cerebellar entrainment. Because stimulation frequencies were derived from baseline gait that was slightly slower than performance in later trials (Table 3), stimulation may have been marginally detuned from participants’ intrinsic locomotor rhythm. However, mixed-effects analyses across trial order revealed no systematic temporal drift (Table S6), indicating that this mismatch did not reflect progressive gait adaptation. Baseline normalization was therefore unlikely to introduce order-related bias. The present study relied on waveform simulation from stimulation parameters, with indirect confirmation through the tACS artifact at Oz; future studies should incorporate direct analog output recording. Post-session ratings indicated that 50 Hz stimulation was more readily perceived than the other active conditions (Fig. 5), although it was not associated with increased discomfort or fatigue. This perceptual salience warrants caution when interpreting the corresponding behavioral effects, because expectancy-related contributions cannot be excluded entirely. At the same time, the absence of increased adverse effects and the selective association with spatial rather than temporal gait measures suggest that the observed effects are unlikely to be explained by expectancy-related mechanisms [63–65].

Future studies should systematically compare unilateral and bihemispheric montages, implement adaptive or closed-loop frequency control, and evaluate multi-session protocols in older adults or neurologically impaired populations. Concurrent behavioral and neurophysiological measures (e.g., EEG coherence, cerebellar-brain inhibition, corticospinal excitability) will be essential to link behavioral effects to underlying neural mechanisms. Furthermore, incorporating surrogate-control synchrony metrics (e.g., phase-scrambled stimulation waveforms or random-phase controls) would help dissociate genuine stimulation-driven synchronization from intrinsic rhythmicity, thereby clarifying whether observed temporal alignment reflects true entrainment or resonance with endogenous dynamics [27, 66].

Although gait-matched stimulation did not yield entrainment-specific effects beyond sham, the dissociation between gamma-related spatial effects and low-frequency phase alignment may still inform the development of frequency-tailored stimulation strategies for gait rehabilitation [30, 31]. Gamma-band stimulation warrants further investigation in gait disorders characterized by reduced gait speed, reduced stride length, or impaired propulsion, including some post-stroke locomotor deficits and aspects of Parkinsonian gait [67, 68]. By contrast, cerebellar ataxia is more typically characterized by instability and increased gait variability [69, 70], suggesting that frequency-matched protocols may be more relevant for probing temporal gait control than for targeting spatial gait output in such populations. Applying frequency-matched c-tACS in cerebellar or cortico-cerebellar disorders could further help determine whether stimulation can induce entrainment-like coupling beyond naturally occurring phase alignment. The modest increase in phase alignment during gait initiation (iGCF-10%) further suggests that locomotor transitions may represent periods of heightened sensitivity to rhythmic input, consistent with the distinct anticipatory control demands of gait initiation [56] and supporting further exploration of transition-locked or gait-synchronized stimulation designs [59, 71].

Overall, these findings suggest that rhythmic neuromodulation can interact with cerebellar locomotor networks in frequency-dependent ways. Rigorous multi-session, patient-centered, and electrophysiologically informed studies will be required to determine the neural mechanisms and clinical relevance of these frequency-specific effects.

## Supplementary Information

Below is the link to the electronic supplementary material.

Supplementary Material 1 (DOCX 2.69 MB)

## Data Availability

The datasets, analysis and code generated during this study are available from the corresponding author upon reasonable request.
