# Supplementary material for: Cerebellar Transcranial Alternating Current Stimulation: Frequency-Specific Modulation of Human Gait
Source: Cerebellum. 2026 Jul 20;25(4):111. doi: 10.1007/s12311-026-02037-8 (PMC13385040; doi:10.1007/s12311-026-02037-8)
Supplement: Supplementary file 1 — Supplementary Material 1 (DOCX 2.69 MB) [file 12311_2026_2037_MOESM1_ESM.docx]

Supplementary information

Figure S1: Exemplary segmentation, data processing and alignment with tACS waveform for stop-and-go.

**a** Example of automatic segmentation for Subject 15, Condition 2. The accelerometer signal (blue trace) shows periodic gait-related oscillations. Detected high peaks (red triangles) and low peaks (green inverted triangles) are used to identify step cycles based on adaptive high and low amplitude thresholds (red and green dashed lines, respectively). Vertical dashed lines indicate automatically detected start and end points of consecutive stop-and-go segments. The algorithm identifies valid walking intervals by requiring a minimum number of high peaks separated by low-peak-defined pauses. **b** Example of gait-acceleration preprocessing for Subject 15, Condition 2. Raw accelerometer data (light blue dotted line) were band-pass filtered (dark blue dashed line), and heel-strike events were used to reconstruct a continuous sinusoidal representation of the gait cycle (black solid line). Left (L) and right (R) step markers (red vertical lines) were identified from gait events. The corresponding transcranial alternating current stimulation (tACS) waveform (yellow line) was aligned with the reconstructed gait signal to assess phase synchrony between stimulation and gait dynamics.


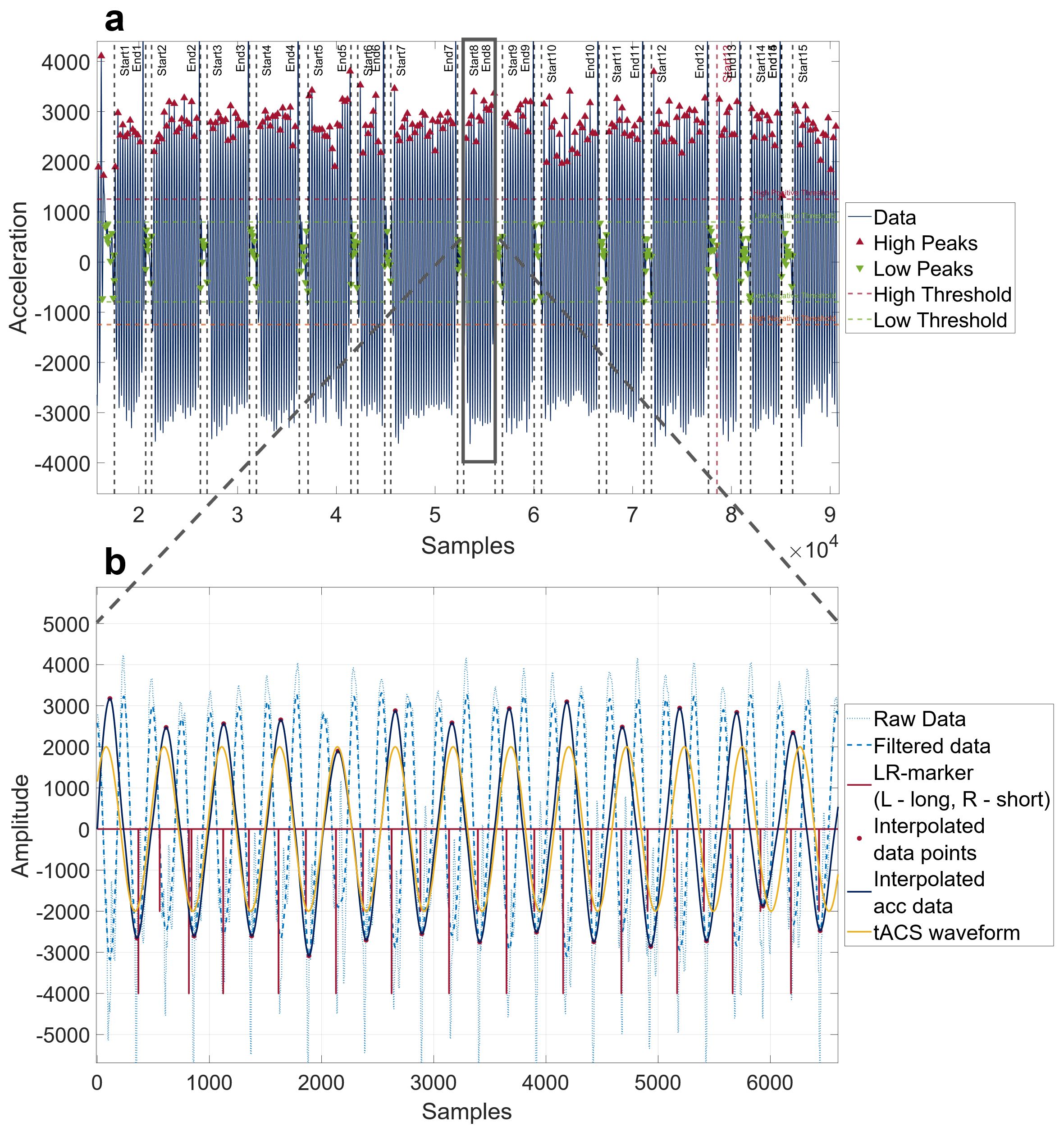


Figure S 2: Bin-wise time course of debiased phase-locking value (dPLV) during the 5-min continuous-walking block across all stimulation conditions.

dPLV was computed in successive non-overlapping bins of five gait cycles and plotted across relative bin order within the stimulation period. The figure illustrates the temporal evolution of stimulation–gait phase alignment during steady-state walking and was used for descriptive inspection of potential within-block changes in online c-tACS effects. No clear systematic drift in dPLV was observed across the stimulation period.


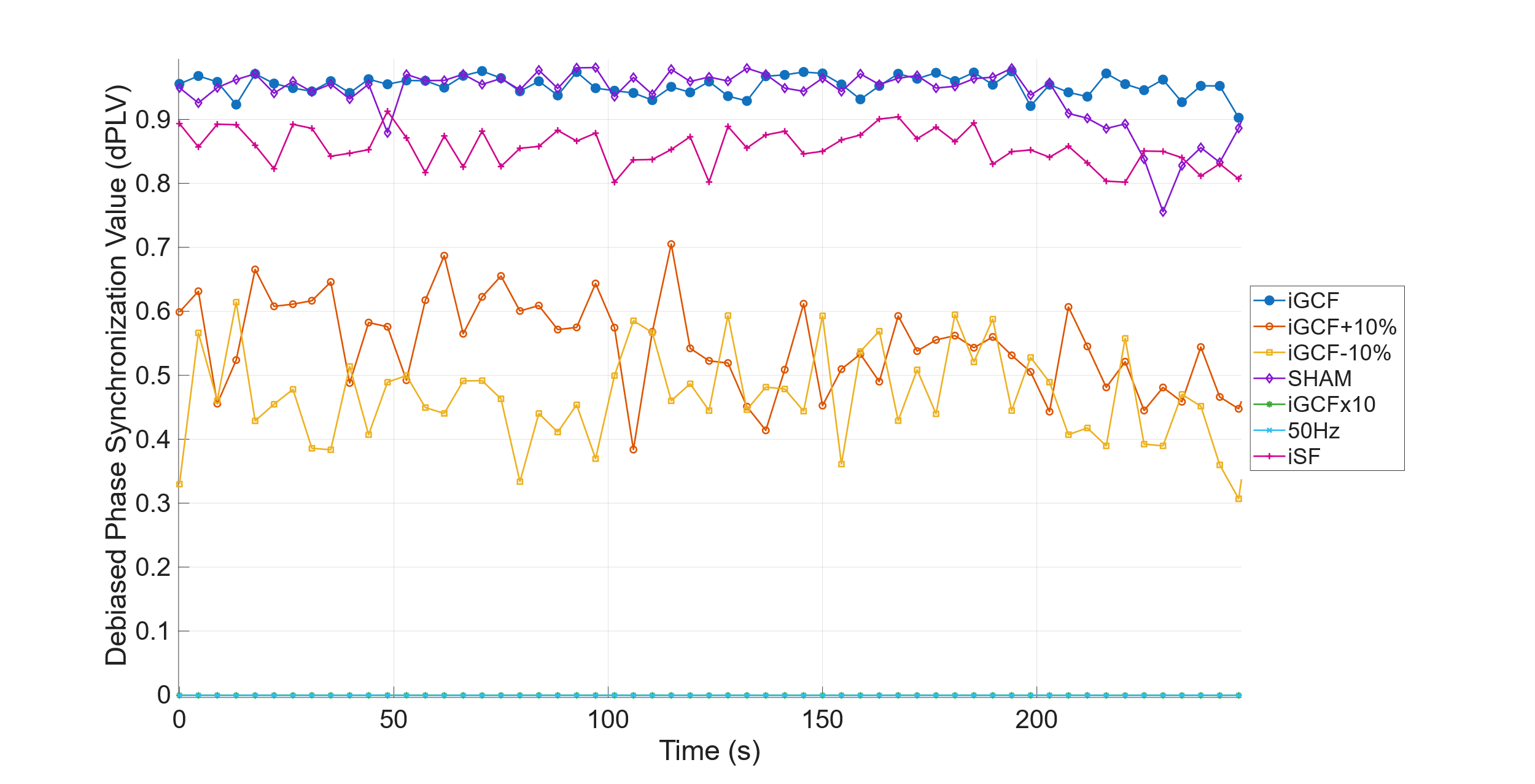


Note. Post hoc comparisons are presented only for significant or interpretively relevant ANOVAs (see main text). Differences represent pairwise contrasts as specified below. “Lower” and “Upper” denote the 95% confidence interval bounds of the mean difference. Bolded values indicate significant contrasts at α = .05. *Italicized k* denotes trend-level comparisons (0.05 < p < 0.10).

Table S1: Tukey-Kramer-corrected post hoc comparisons of Δgait velocity during continuous walking.

Differences are computed as *Condition_1 – Condition_2* (Δvelocity, m/s). Positive values indicate higher gait velocity during *Condition_1*. Results correspond to the significant main effect of Condition in the repeated-measures ANOVA (p < .05). Cohen’s d denotes standardized mean differences.

| **Condition_1** | **Condition_2** | **Difference** | **SE** | **Lower** | **Upper** | ***p*-value** | **Cohen‘s *d*** |
| --- | --- | --- | --- | --- | --- | --- | --- |
| **SHAM** | **50Hz** | **-0.043** | **0.012** | **-0.086** | **0.000** | **0.049** | **0.568** |
| SHAM | iGCF | 0.018 | 0.014 | -0.032 | 0.068 | 0.851 | 0.225 |
| SHAM | iSF | -0.019 | 0.011 | -0.058 | 0.020 | 0.624 | 0.265 |
| SHAM | -10% | 0.014 | 0.013 | -0.032 | 0.059 | 0.929 | 0.184 |
| SHAM | +10% | 0.011 | 0.013 | -0.035 | 0.056 | 0.976 | 0.140 |
| SHAM | x10 | -0.030 | 0.011 | -0.070 | 0.010 | 0.200 | 0.418 |
| **50Hz** | **iGCF** | **0.061** | **0.013** | **0.016** | **0.105** | **0.007** | **0.759** |
| 50Hz | iSF | 0.024 | 0.009 | -0.008 | 0.057 | 0.196 | 0.342 |
| **50Hz** | **-10%** | **0.056** | **0.008** | **0.028** | **0.084** | **<0.001** | **0.765** |
| **50Hz** | **+10%** | **0.053** | **0.012** | **0.011** | **0.096** | **0.012** | **0.705** |
| 50Hz | x10 | 0.013 | 0.006 | -0.008 | 0.034 | 0.363 | 0.184 |
| iGCF | iSF | -0.037 | 0.017 | -0.097 | 0.024 | 0.386 | 0.485 |
| iGCF | -10% | -0.004 | 0.012 | -0.049 | 0.040 | 1.000 | 0.057 |
| iGCF | +10% | -0.007 | 0.010 | -0.042 | 0.027 | 0.985 | 0.092 |
| iGCF | x10 | -0.048 | 0.015 | -0.102 | 0.007 | 0.101 | 0.626 |
| iSF | -10% | 0.032 | 0.014 | -0.018 | 0.082 | 0.323 | 0.469 |
| iSF | +10% | 0.029 | 0.015 | -0.024 | 0.082 | 0.482 | 0.413 |
| iSF | x10 | -0.011 | 0.007 | -0.034 | 0.012 | 0.636 | 0.167 |
| -10% | +10% | -0.003 | 0.011 | -0.042 | 0.036 | 1.000 | 0.040 |
| **-10%** | **x10** | **-0.043** | **0.010** | **-0.080** | **-0.007** | **0.017** | **0.624** |
| +10% | x10 | -0.040 | 0.012 | -0.084 | 0.003 | 0.074 | 0.564 |

Table S2: Tukey-Kramer-corrected post hoc comparisons of Δstride length during continuous walking.
Differences are computed as *Condition_1 – Condition_2* (Δstride length, cm). Positive values indicate longer stride length during *Condition_1*. Post hoc comparisons correspond to the significant main effect of Condition in the repeated-measures ANOVA. Cohen’s d represents standardized effect sizes.

| **Condition_1** | **Condition_2** | **Difference** | **SE** | **Lower** | **Upper** | ***p*-value** | **Cohen‘s *d*** |
| --- | --- | --- | --- | --- | --- | --- | --- |
| **SHAM** | **50Hz** | **-4.200** | **0.991** | **-7.876** | **-0.524** | **0.024** | **0.771** |
| SHAM | iGCF | 0.682 | 1.083 | -3.335 | 4.700 | 0.994 | 0.134 |
| SHAM | iSF | -1.628 | 0.776 | -4.506 | 1.250 | 0.425 | 0.290 |
| SHAM | -10% | -0.022 | 0.656 | -2.454 | 2.409 | 1.000 | 0.004 |
| SHAM | +10% | 0.152 | 0.990 | -3.520 | 3.825 | 1.000 | 0.029 |
| SHAM | x10 | -2.981 | 0.910 | -6.356 | 0.395 | 0.092 | 0.543 |
| **50Hz** | **iGCF** | **4.882** | **0.832** | **1.796** | **7.968** | **0.003** | **1.057** |
| 50Hz | iSF | 2.572 | 0.779 | -0.316 | 5.460 | 0.088 | 0.495 |
| **50Hz** | **-10%** | **4.177** | **0.771** | **1.317** | **7.038** | **0.005** | **0.792** |
| **50Hz** | **+10%** | **4.352** | **0.925** | **0.923** | **7.781** | **0.013** | **0.924** |
| 50Hz | x10 | 1.219 | 0.503 | -0.646 | 3.084 | 0.288 | 0.241 |
| iGCF | iSF | -2.310 | 1.049 | -6.199 | 1.579 | 0.377 | 0.480 |
| iGCF | -10% | -0.705 | 0.952 | -4.233 | 2.824 | 0.986 | 0.144 |
| iGCF | +10% | -0.530 | 0.849 | -3.677 | 2.617 | 0.994 | 0.123 |
| iGCF | x10 | -3.663 | 1.011 | -7.410 | 0.085 | *0.056* | 0.783 |
| iSF | -10% | 1.605 | 0.811 | -1.401 | 4.611 | 0.483 | 0.295 |
| iSF | +10% | 1.780 | 1.001 | -1.932 | 5.493 | 0.590 | 0.363 |
| iSF | x10 | -1.353 | 0.806 | -4.343 | 1.637 | 0.644 | 0.258 |
| -10% | +10% | 0.175 | 0.924 | -3.253 | 3.602 | 1.000 | 0.035 |
| **-10%** | **x10** | **-2.958** | **0.745** | **-5.720** | **-0.196** | **0.035** | **0.556** |
| **+10%** | **x10** | **-3.133** | **0.646** | **-5.527** | **-0.739** | **0.010** | **0.658** |

Table S3: Exploratory Tukey-Kramer-corrected post hoc comparisons of Δgait-initiation time during the stop-and-go task.

Differences are computed as *Condition_1 – Condition_2* (Δinitiation time, ms). Positive values indicate longer initiation times during *Condition_1*. Displayed for exploratory interpretation of the Condition effect (F_6, 60_ = 2.32, *p* = 0.121, $\eta_{p}^{2}$ = 0.19). Cohen’s d values are reported for descriptive completeness.

| **Condition_1** | **Condition_2** | **Difference** | **SE** | **Lower** | **Upper** | ***p*-value** | **Cohen‘s *d*** |
| --- | --- | --- | --- | --- | --- | --- | --- |
| SHAM | 50Hz | -2.400 | 9.148 | -35.545 | 30.745 | 1.000 | 0.072 |
| SHAM | iGCF | 15.636 | 9.678 | -19.430 | 50.703 | 0.677 | 0.446 |
| SHAM | iSF | 7.200 | 8.706 | -24.346 | 38.746 | 0.976 | 0.213 |
| SHAM | -10% | 2.145 | 8.478 | -28.572 | 32.863 | 1.000 | 0.060 |
| SHAM | +10% | 13.636 | 10.879 | -25.783 | 53.056 | 0.858 | 0.477 |
| SHAM | x10 | 0.545 | 7.868 | -27.964 | 29.055 | 1.000 | 0.017 |
| 50Hz | iGCF | 18.036 | 12.972 | -28.965 | 65.037 | 0.796 | 0.484 |
| 50Hz | iSF | 9.600 | 8.053 | -19.580 | 38.780 | 0.882 | 0.266 |
| 50Hz | -10% | 4.545 | 12.881 | -42.127 | 51.218 | 1.000 | 0.120 |
| 50Hz | +10% | 16.036 | 14.023 | -34.775 | 66.848 | 0.900 | 0.514 |
| 50Hz | x10 | 2.945 | 10.063 | -33.517 | 39.407 | 1.000 | 0.084 |
| iGCF | iSF | -8.436 | 12.684 | -54.394 | 37.521 | 0.992 | 0.224 |
| iGCF | -10% | -13.491 | 3.564 | -26.404 | -0.578 | *0.039* | 0.343 |
| iGCF | +10% | -2.000 | 10.660 | -40.624 | 36.624 | 1.000 | 0.061 |
| iGCF | x10 | -15.091 | 11.085 | -55.257 | 25.075 | 0.810 | 0.412 |
| iSF | -10% | -5.055 | 12.110 | -48.934 | 38.825 | 0.999 | 0.132 |
| iSF | +10% | 6.436 | 11.203 | -34.155 | 47.027 | 0.996 | 0.202 |
| iSF | x10 | -6.655 | 12.311 | -51.262 | 37.952 | 0.997 | 0.187 |
| -10% | +10% | 11.491 | 10.829 | -27.745 | 50.727 | 0.926 | 0.341 |
| -10% | x10 | -1.600 | 10.455 | -39.482 | 36.282 | 1.000 | 0.043 |
| +10% | x10 | -13.091 | 10.551 | -51.320 | 25.139 | 0.863 | 0.429 |

Table S4: Holm-Bonferroni-corrected post hoc comparisons of the debiased phase-locking value (dPLV) during continuous walking and stop-and-go tasks.

Differences are computed as *Condition_1 – Condition_2* (dPLV units). Positive values indicate stronger phase alignment during *Condition_1*, and negative values stronger synchronization during *Condition_2*. Results correspond to the main effect of Condition and exploratory Task × Condition contrasts from the two-way repeated-measures ANOVA. Cohen’s *d* represents standardized mean differences. The upper section (“Walking”) refers to continuous-walking trials; the lower section (“Stop-and-go”) to intermittent start–stop trials. Higher dPLV values reflect stronger phase alignment between the stimulation waveform and gait acceleration.

| **Task** | **Condition_1** | **Condition_2** | **Difference** | **SE** | **Lower** | **Upper** | ***p*-value** | **Cohen's *d*** |
| --- | --- | --- | --- | --- | --- | --- | --- | --- |
| **Walking** | **iGCF** | **+10%** | **0.413** | **0.054** | **0.000** | **0.227** | **0.002** | **20.250** |
|  | **iGCF** | **-10%** | **0.493** | **0.025** | **0.000** | **0.406** | **<0.001** | **51.764** |
|  | iGCF | SHAM | -0.008 | 0.007 | 0.850 | -0.031 | 5.882 | -3.382 |
|  | **iGCF** | **x10** | **0.961** | **0.013** | **0.000** | **0.917** | **<0.001** | **198.705** |
|  | **iGCF** | **50Hz** | **0.961** | **0.013** | **0.000** | **0.917** | **<0.001** | **198.716** |
|  | iGCF | iSF | 0.094 | 0.032 | 0.116 | -0.016 | 1.395 | 7.811 |
|  | +10% | -10% | 0.080 | 0.065 | 0.873 | -0.146 | 4.251 | 3.235 |
|  | **+10%** | **SHAM** | **-0.422** | **0.052** | **0.000** | **-0.601** | **0.001** | **-21.463** |
|  | **+10%** | **x10** | **0.548** | **0.045** | **0.000** | **0.393** | **<0.001** | **32.291** |
|  | **+10%** | **50Hz** | **0.548** | **0.045** | **0.000** | **0.393** | **<0.001** | **32.289** |
|  | +10% | iSF | -0.320 | 0.073 | 0.010 | -0.573 | 0.244 | -11.557 |
|  | **-10%** | **SHAM** | **-0.502** | **0.025** | **0.000** | **-0.588** | **<0.001** | **-53.509** |
|  | **-10%** | **x10** | **0.468** | **0.025** | **0.000** | **0.381** | **<0.001** | **49.378** |
|  | **-10%** | **50Hz** | **0.468** | **0.025** | **0.000** | **0.381** | **<0.001** | **49.376** |
|  | **-10%** | **iSF** | **-0.400** | **0.021** | **0.000** | **-0.472** | **<0.001** | **-50.483** |
|  | **SHAM** | **x10** | **0.970** | **0.011** | **0.000** | **0.933** | **<0.001** | **244.181** |
|  | **SHAM** | **50Hz** | **0.970** | **0.011** | **0.000** | **0.933** | **<0.001** | **244.188** |
|  | SHAM | iSF | 0.102 | 0.034 | 0.103 | -0.014 | 1.441 | 8.007 |
|  | x10 | 50Hz | 0.000 | 0.000 | 0.482 | 0.000 | 4.818 | -5.175 |
|  | **x10** | **iSF** | **-0.868** | **0.033** | **0.000** | **-0.983** | **<0.001** | **-68.969** |
|  | **50Hz** | **iSF** | **-0.868** | **0.033** | **0.000** | **-0.983** | **<0.001** | **-68.970** |
|  |  |  |  |  |  |  |  |  |
| **Stop-and-go** | **iGCF** | **+10%** | **0.410** | **0.032** | **0.000** | **0.299** | **<0.001** | **33.843** |
|  | iGCF | -10% | 0.199 | 0.038 | 0.002 | 0.068 | 0.057 | 13.938 |
|  | iGCF | SHAM | -0.006 | 0.013 | 0.999 | -0.051 | 2.620 | -1.204 |
|  | **iGCF** | **x10** | **0.933** | **0.016** | **0.000** | **0.879** | **<0.001** | **157.348** |
|  | **iGCF** | **50Hz** | **0.933** | **0.016** | **0.000** | **0.879** | **<0.001** | **157.200** |
|  | iGCF | iSF | 0.048 | 0.011 | 0.015 | 0.008 | 0.335 | 10.948 |
|  | +10% | -10% | -0.212 | 0.063 | 0.057 | -0.428 | 1.028 | -8.933 |
|  | **+10%** | **SHAM** | **-0.416** | **0.034** | **0.000** | **-0.532** | **<0.001** | **-32.737** |
|  | **+10%** | **x10** | **0.523** | **0.041** | **0.000** | **0.380** | **<0.001** | **33.422** |
|  | **+10%** | **50Hz** | **0.523** | **0.041** | **0.000** | **0.380** | **<0.001** | **33.376** |
|  | **+10%** | **iSF** | **-0.363** | **0.032** | **0.000** | **-0.474** | **<0.001** | **-29.610** |
|  | **-10%** | **SHAM** | **-0.205** | **0.037** | **0.002** | **-0.333** | **0.043** | **-14.517** |
|  | **-10%** | **x10** | **0.735** | **0.028** | **0.000** | **0.637** | **<0.001** | **68.809** |
|  | **-10%** | **50Hz** | **0.735** | **0.028** | **0.000** | **0.637** | **<0.001** | **68.682** |
|  | -10% | iSF | -0.151 | 0.039 | 0.024 | -0.285 | 0.473 | -10.279 |
|  | **SHAM** | **x10** | **0.939** | **0.014** | **0.000** | **0.892** | **<0.001** | **182.549** |
|  | **SHAM** | **50Hz** | **0.939** | **0.014** | **0.000** | **0.892** | **<0.001** | **182.086** |
|  | SHAM | iSF | 0.054 | 0.016 | 0.071 | -0.003 | 1.138 | 8.591 |
|  | x10 | 50Hz | 0.000 | 0.000 | 0.840 | -0.001 | 6.723 | -3.440 |
|  | **x10** | **iSF** | **-0.886** | **0.020** | **0.000** | **-0.956** | **<0.001** | **-114.570** |
|  | **50Hz** | **iSF** | **-0.886** | **0.020** | **0.000** | **-0.956** | **<0.001** | **-114.413** |

Table S5: Exploratory Holm-Bonferroni-corrected post hoc comparisons of task-related differences in the debiased phase-locking value (dPLV) between continuous walking and stop-and-go conditions.

Differences are computed as *Stop-and-go – Gait* (dPLV units). Positive values indicate stronger phase-locking during the stop-and-go task, and negative values indicate greater synchronization during continuous walking. Cohen’s *d* represents standardized mean differences. Results reflect within-protocol contrasts between continuous-walking (Gait) and stop-and-go (Stop-and-go) tasks.

| **Protocol** | **Task_1** | **Task_2** | **Difference** | **SE** | **Lower** | **Upper** | ***p*-value** | **Cohen's *d*** |
| --- | --- | --- | --- | --- | --- | --- | --- | --- |
| iGCF | Gait | Stop-and-go | 0.028 | 0.022 | -0.020 | 0.076 | 1.804 | 3.367 |
| plusTen | Gait | Stop-and-go | 0.025 | 0.025 | -0.029 | 0.078 | 2.008 | 2.651 |
| **minusTen** | **Gait** | **Stop-and-go** | **-0.267** | **0.022** | **-0.314** | **-0.220** | **0.000** | **-32.617** |
| SHAM | Gait | Stop-and-go | 0.030 | 0.019 | -0.010 | 0.071 | 1.288 | 4.291 |
| **xTen** | **Gait** | **Stop-and-go** | **0.000** | **0.000** | **0.000** | **0.000** | **0.015** | **10.897** |
| fiftyHz | Gait | Stop-and-go | 0.000 | 0.000 | 0.000 | 0.000 | 1.674 | -2.352 |
| iSF | Gait | Stop-and-go | -0.018 | 0.043 | -0.110 | 0.074 | 1.355 | -1.125 |

Table S6: Summary of linear mixed-effect model parameters for continuous walking metrics across trial order.

|  | **Slope**  **(β)** | **SE** | **t-Stat** | **Df** | ***p*-value** | **Lower** | **Upper** | **Adjusted R^2^** | **RandomSD Intercept** | **RandomSD Time** | **Residual SD** | **AIC** | **BIC** |
| --- | --- | --- | --- | --- | --- | --- | --- | --- | --- | --- | --- | --- | --- |
| Gait velocity | 0.001 | 0.002 | 0.499 | 94 | 0.619 | -0.004 | 0.006 | 0.886 | 0.099 | 0.005 | 0.041 | -274.341 | -258.955 |
| Cadence | 0.040 | 0.062 | 0.638 | 110 | 0.525 | -0.083 | 0.163 | 0.935 | 5.185 | 0.067 | 1.441 | 477.600 | 493.911 |
| Stride length | 0.001 | 0.002 | 0.705 | 78 | 0.483 | -0.002 | 0.004 | 0.924 | 0.087 | 0.002 | 0.029 | -280.588 | -266.296 |

Linear mixed-effects models examined temporal trends in gait velocity, cadence, and stride length across experimental blocks, with **Time** as a fixed effect and random intercepts and slopes per subject (*Gait parameter ~ 1 + Time + (1 + Time | Subject)*). Reported are fixed-effect slopes (β ± standard error), *t*- and *p*-values, 95% confidence intervals (CIs), adjusted R², random and residual standard deviations (SDs), and model fit indices (AIC, BIC). None of the parameters showed a significant time effect, indicating stable gait performance and the absence of systematic spatio-temporal adaptation.

Power analysis and sensitivity estimation

An *a priori* power analysis was conducted using G*Power 3.1.9.7 (Faul et al., 2007) for a repeated-measures ANOVA (within-subject factor). The expected effect size was derived from Koganemaru et al. (2020), who reported a significant frequency-dependent effect of cerebellar transcranial alternating current stimulation (tACS) on gait modulation (*F*(2,26) = 3.45, *p* = .047; partial η² = 0.21; *f* = 0.51).

Assuming α = .05, desired power (1–β) = .95, correlation among repeated measures = 0.5, nonsphericity correction ε = 1, and seven within-subject stimulation conditions, the required total sample size was *N* = 7.

For the actual sample size (*N* = 15), a sensitivity analysis using the same parameters indicated a minimum detectable effect size of *f* ≥ 0.29 (ηp² ≥ 0.08) at 80% power, corresponding to medium-to-large within-subject effects.

Because Koganemaru et al. (2020) employed a three-level design, the observed effect size was conservatively extrapolated to the present seven-level design under the assumption that frequency-dependent modulation of gait would be of comparable magnitude.
